# Burden of Disease Methods: A Guide to Calculate COVID-19 Disability-Adjusted Life Years

**DOI:** 10.3389/ijph.2021.619011

**Published:** 2021-03-05

**Authors:** Grant M. A. Wyper, Ricardo M. A. Assunção, Edoardo Colzani, Ian Grant, Juanita A. Haagsma, Giske Lagerweij, Elena Von der Lippe, Scott A. McDonald, Sara M. Pires, Michael Porst, Niko Speybroeck, Brecht Devleesschauwer

**Affiliations:** ^1^ Place and Wellbeing Directorate, Public Health Scotland, Glasgow, Scotland; ^2^ Food and Nutrition Department, National Institute of Health Dr. Ricardo Jorge, Lisbon, Portugal; ^3^ European Centre for Disease Prevention and Control, Stockholm, Sweden; ^4^ Data Driven Innovation Directorate, Public Health Scotland, Edinburgh, Scotland; ^5^ Department of Public Health, Erasmus MC University Medical Center, Rotterdam, Netherlands; ^6^ Centre for Infectious Disease Control, National Institute for Public Health and the Environment (RIVM), Bilthoven, Netherlands; ^7^ Department of Epidemiology and Health Monitoring, Robert Koch Institute, Berlin, Germany; ^8^ National Food Institute, Technical University of Denmark, Lyngby, Denmark; ^9^ Research Institute of Health and Society, Catholic University of Louvain, Brussels, Belgium; ^10^ Department of Epidemiology and Public Health, Sciensano, Brussels, Belgium; ^11^ Department of Veterinary Public Health and Food Safety, Ghent University, Merelbeke, Belgium

**Keywords:** COVID-19, disability-adjusted life year, years lived with disability, years of life lost, coronavirus, European burden of disease network

## Background

To date, most efforts to understand the comparative population health impact of COVID-19 have been made using mortality-based metrics [1,2]. This has intensified discussion over methodological choices; in particular, how we value the life-years prematurely lost due to COVID-19 [3]. So far, the direct impact of COVID-19 on population health has varied across countries, with wide variation in incidence and infection fatality rates [4]. Understanding and quantifying the combined impact of morbidity and mortality is a key step to standardizing comparisons across countries, and to quantify the within-country impact of COVID-19 relative to other causes of disease and injury, sub-national areas or demographics [5]. This can be achieved by estimating summary measures of population health like disability-adjusted life years (DALYs). The estimation of DALYs is useful to provide comprehensive and comparative public health intelligence to inform decision-making for the management of the COVID-19 pandemic, particularly around the extent of direct and indirect consequences [6]. At present, the Global Burden of Disease (GBD) study has not integrated COVID-19. Some studies have already estimated DALYs due to COVID-19. The first published assessment was performed for Korea, up until the end of April 2020 [7]. An assessment, using a similar time frame, followed for Italy [8]. To date, published studies have only included one COVID-19 related health state, or disability weights were country-specific [7–9].

## Aim

Our paper provides a step-by-step guide to define COVID-19 as a cause of disease burden, which can be used to calculate DALYs. Additionally, we suggest pragmatic data inputs, reflecting that availability and quality of data inputs will vary by country. This paper builds on previous DALY calculation guides [10,11]. As our paper provides suggestions for different solutions, we recommend that users should be clear about their methodological choices to aid comparisons and knowledge translation.

## Methodology

The impact of COVID-19 on health occurs through two main pathways: directly, as an infectious disease; and indirectly, as a risk factor, for example, through increases in mental health issues due to national lockdowns or delays to surgery, follow-ups and diagnoses through restrictions to vital healthcare services [6]. A group within COST Action (European Cooperation in Science and Technology) CA18218—European Burden of disease Network—convened to establish a common methodological approach to estimate DALYs directly due to COVID-19.

This paper presents the developed consensus approach in three steps. The first relates to defining study parameters. The steps that follow relate to estimating the impact of morbidity, in terms of years lived with disability (YLD); and mortality, in terms of years lost to premature mortality (YLL). DALYs quantify the full population health impact and are calculated by summing YLD and YLL. DALYs can be estimated based on grouped characteristics of interest, such as demographics (e.g. age, sex, socioeconomic status, and ethnicity), geographical region, or time.

### Step 1: Defining Study Parameters

The first step in estimating DALYs is to define the space and time for which the burden of disease assessment will take place. This involves clear definitions of the population, which is being studied, for example a country, or sub-national region of a country. The time period is usually defined as a specific year or grouped years. However as COVID-19 is a new disease, only shorter time periods will initially be available.

### Step 2: Morbidity—Estimating YLD

**FIGURE 1:**
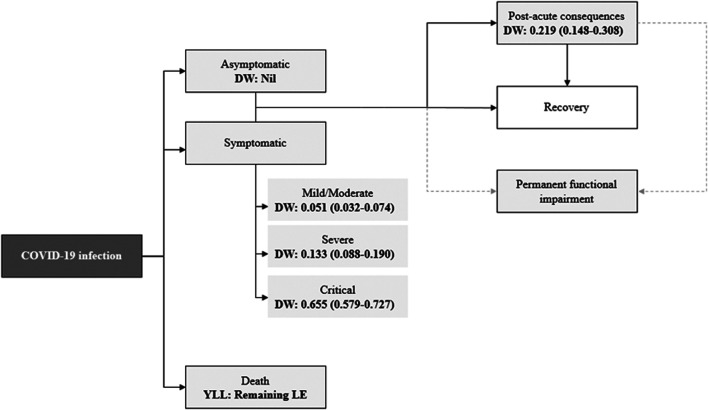
COVID-19 outcomes model and value of health loss. “DW” denotes disability weight; 95% uncertainty intervals for DW are displayed in brackets; “YLL” denotes years of life lost due to premature mortality; “LE” denotes life expectancy; dashed line represents that individuals suffering from COVID-19 may suffer permanent impairments from other health states.

The second step in defining morbidity due to COVID-19 is to establish the direct health states, which are experienced by individuals infected by COVID-19 in an incidence-based model. For each health state, there are three parameters, which require data inputs: incidence; duration; and disability. YLD are calculated by summing the product of the number of cases, duration (in years) and disability weight, across all health states:

$$\text{YLD}=\sum_{h=1}^{l}{\text{YLD}}_{h}=\sum_{h=1}^{l}\text{Number}\;\text{of}\;{\text{cases}}_{h}\times {\text{duration}}_{h}\times \text{disability}\;{\text{weight}}_{h},$$

with *h* = health state and *l* = number of health states. The COVID-19 outcomes model is outlined in Figure 1, with detailed information on health states, data input proxies and corresponding disability weights outlined in Table 1. Health state names, descriptions and disability weights are currently based on those from the GBD 2019 study for infectious diseases of the lower respiratory tract, with the exception of those requiring intensive care, which was defined by the European Disability Weight study [12–14]. As new evidence on type, severity and duration of COVID-19 sequelae emerges, the outcome tree should be updated accordingly.

**TABLE 1:** Example of health states and disability weights to consider for estimating the burden of COVID-19.

Name	Description	Data input proxy	Disability weight (95%uncertainty interval)	Source
Asymptomatic	Has infection but experiences no symptoms	Estimates of suspected asymptomatic community cases	Nil	
Moderate (community, seeking healthcare assistance)	Has a fever and aches, and feels weak, which causes some difficulty with daily activities	Positive/suspected community cases	0.051 (0.032–0.074)	Salomon et al. (2015)
Severe (hospitalized, non-intensive care)	Has a high fever and pain, and feels very weak, which causes great difficulty with daily activities	Positive/suspected cases requiring a non-intensive care hospitalization	0.133 (0.088–0.190)	Salomon et al. (2015)
Critical (hospitalized, intensive care)	Intensive care unit admission (with or without respiratory support)	Positive/suspected cases requiring intensive care hospitalization	0.655 (0.579–0.727)	Haagsma et al. (2015)
Post-acute consequences (fatigue, emotional lability, insomnia)	Is always tired and easily upset. The person feels pain all over the body and is depressed	Transition probabilities from cases to post-acute consequences	0.219 (0.148–0.308)	Salomon et al. (2015)

Data inputs for YLD are largely dependent on the timeliness and availability of estimates reported by individual countries, and are sometimes based on COVID-19 transmission models. Our model should therefore be adapted to suit individual circumstances, for example, to reflect differences in restrictions and triaging across hospitals, regions and countries, and how the state of the pandemic increases service pressure, affecting the availability of healthcare services. This recognizes the fact that the distribution of disease occurrence across health states is likely to vary by location [15].

By definition, acute infection occurrence only contributes to YLD if cases are symptomatic, however patients that are initially asymptomatic may suffer YLD through post-acute consequences (Figure 1). Although those that have sought healthcare support, or testing, will vary between countries testing regimes and healthcare capacity, identification of suspected/positive cases is the most widely available proxy to use for symptomatic cases. There are potential issues with the misclassification of asymptomatic cases that are tested, due to difficulties capturing data on non-hospitalized symptomatic cases. However, we cannot yet quantify the extent of this bias. Corrective factors can be applied as, and when, this information emerges. People who tested positive and/or notified cases represent only a fraction of the true number of community cases (under-ascertainment) as an unknown proportion do not seek healthcare, and even among persons with severe or critical disease (hospitalized or in intensive care) patient counts could be subject to underreporting. As with most other infectious diseases, for accurate estimation of YLD due to COVID-19, adjustment of observed case counts for both under-ascertainment and underreporting is fundamental. A number of methods are available to adjust input data to estimate the true incidence [16]. There is very little research to date on how this estimation can be achieved for COVID-19. Data on healthcare-seeking behavior and incidence estimates from transmission models are two possible routes, if they are available for the country/region in question. If only recorded case/patient counts are used in the YLD calculation, then between-country comparisons must recognize the inherent limitations due to differences in surveillance systems, testing policy, and case management. It is therefore important to capture the uncertainty around estimates, particularly if referring to country comparisons. Adjustment factors, such as positive tests as a proportion of total swab tests could be used to adjust for variations in testing rates across countries and over time to standardize comparisons. However, it is important to establish the context of testing policies when making comparisons as they may differ. For example, there may be locational differences in testing policies, such as focusing testing on high-risk settings, or as part of contact-tracing.

Assumptions over the mean duration in a health state can be replaced by the calculation of person-years of infection when daily data have been reported. When these cannot be calculated, it is preferred to use estimates of the mean duration of 7.79 days (95% uncertainty interval: 6.20–9.64 days) for lower respiratory tract infections from the GBD study, or to derive them from consultation with within-country clinical expert groups [17]. Additionally, if the granularity of data for community cases permits, the YLD disease model can be refined to include mild cases using a disability weight of 0.006 (95% uncertainty interval: 0.002–0.012) from the upper respiratory tract infections model.

There are several options to incorporate uncertainty in YLD estimates. Credible intervals and/or lower and upper limits of occurrence, duration, and disability weights can be used. These can also include utilizing corrective factors, as evidence emerges, regarding any issues with selection bias of input data. Additional scenario analyses can also be undertaken to illustrate that other plausible scenarios may have small effects on overall DALYs, since YLD accumulates at a slower rate than YLL. At present, there have been limited reports on the post-acute, and long-term consequences of COVID-19 [18,19], but as more epidemiological information on the occurrence and duration becomes available, YLD can be refined to include these estimates using appropriate transition probabilities.

Emerging evidence is showing that in some cases post-acute consequences of COVID-19 (e.g. anosmia) may significantly affect quality of life and that sometimes (e.g. persistent symptoms) they may also lead to prolonged healthcare utilization. It is however too early to assess the full spectrum and burden of long-term sequelae associated with COVID-19, so new knowledge on type, duration and disability weight of COVID-19 sequelae will need to be incorporated as more solid evidence becomes available. Under a pathogen-based approach to estimating DALYs, the long-term complications are attributed to the initial infection to represent its natural history, provided there is sufficient evidence for a causal relationship [20,21]. An alternative is to assign any resultant new, or exacerbated, non-communicable disease (NCD) within assessments of cause-specific NCD burden. This is similar to the approach that the GBD study use for causes of disease such as cirrhosis, or liver cancer, caused be hepatitis C infection [22].

Finally, we acknowledge that the COVID-19 crisis has led to wider health effects that go beyond the morbidity linked to the disease as such. For instance, lockdowns have led to an insurgence of mental health problems, delayed health care-seeking behavior and deferred follow-up of chronic conditions may have increased the severity of common chronic non-communicable diseases and their complications. We however recommend to not include these indirect effects in the COVID-19 disease model, and to make an explicit distinction between COVID-19 as a disease, and the COVID-19 crisis as a risk factor for ill-health. As evidence emerges around the direct effects of COVID-19 our disease model may warrant revisions.

### Step 3: Mortality—Estimating YLL

When estimating YLL, two inputs are required: the number of deaths; and conditional life expectancy, both of which must be defined at age-group level. YLL is estimated as:

$$\text{YLL}=\sum_{a=1}^{n}{\text{YLL}}_{a}=\sum_{a=1}^{n}\text{Number}\;\text{of}\;{\text{deaths}}_{a}\times \text{Remaining}\;\text{life}\;{\text{expectancy}}_{a},$$

where *a* = age-group, e.g. 1-year or 5-years group, *n* = number of age-groups. Age-conditional life expectancy can be defined from life tables (e.g. national, regional, or aspirational), and depends on the age group. For internationally comparable results, which may be important during a public health emergency of international concern, the use of an aspirational life table may be preferable [3].

Estimates of the number of deaths will largely depend on data availability. Some countries may have timely mortality data available by mutually exclusive causes of death, in which case the underlying cause of death should be used. Depending on the national death registration policy, some death certificates may indicate ill-defined causes of death. If possible, then redistribution can be considered to refine estimates. Otherwise if deaths with COVID-19 as a contributing cause can be determined, then a sensitivity analysis using COVID-19 related (both COVID-19 cause-specific and contributing) deaths may help gauge the degree of potential underestimation of using cause-specific deaths. Additionally, corrections can be considered to address the potential underestimation of COVID-19 deaths, arising due to death certification issues in the absence of laboratory confirmed tests [23]. If death certification statistics are not available, other alternatives exist. In countries where COVID-19 is a notifiable disease, published mortality data on cases can be used. However, care should be taken to ensure that these data are unlikely to represent non-COVID-19 deaths, for example by assessing the time-based inclusion criteria. Alternatively, providing that non-COVID-19 mortality rates are stable, estimates of the number of excess deaths could be used as a proxy for COVID-19 deaths. An uncertainty in using this approach is that these deaths will also include a proportion due to indirect consequences of COVID-19, for example those that occur as a consequence of closures of vital services during national lockdowns. Another option is to consider using transition probabilities from published literature, which are useful for countries without timely or reliable death registration information, taken from existing case-fatality ratios. However, it may not be the case that these are readily adaptable in different contexts, as the management of patients may vary from one context to another, and similarly the probability of dying given the same disease severity.

## Conclusion

The use of DALYs for estimating the burden of COVID-19 should be further explored in order to quantify the overall impact of the pandemic going well beyond available COVID-19 mortality figures. The use of burden of disease estimates can inform policy makers about the actual magnitude of the overall burden of the COVID-19 pandemic and may help trigger action or better plan suitable mitigating measures.

A standard methodology for DALYs attributable to COVID-19 may allow more accurate and comparable estimates of the burden of this disease. Uncertainties persist around COVID-19 data quality and availability of information concerning sequelae from COVID-19. Transparent reporting of uncertainties and limitations is warranted to favor a correct interpretation of the results.

## Data Availability

The original contributions presented in the study are included in the article. Further inquiries can be directed to the corresponding author.
